# Comparison of LI-RADS v.2017 and ESGAR Guidelines Imaging Criteria in HCC Diagnosis Using MRI with Hepatobiliary Contrast Agents

**DOI:** 10.1155/2018/7465126

**Published:** 2018-07-15

**Authors:** Grzegorz Rosiak, Joanna Podgorska, Edyta Rosiak, Andrzej Cieszanowski

**Affiliations:** ^1^2nd Radiology Department, Warsaw Medical University, SPCSK, ul. Banacha 1a, Warsaw, Poland; ^2^Department of Radiology, Maria Sklodowska-Curie Memorial Cancer Centre, Institute of Oncology, Warsaw, Poland

## Abstract

**Purpose:**

The purpose of this study was to assess and compare diagnostic ability of LI-RADS (LR) v. 2017 and ESGAR guidelines in hepatocellular carcinoma (HCC) diagnosis using MRI with hepatobiliary contrast agents.

**Methods:**

Seventy pathologically confirmed lesions in 32 patients (24 males and 8 females) who had MRI with hepatobiliary contrast done before surgery or biopsy were reviewed retrospectively. Six lesions were <10mm, 31 lesions 10-19mm, and 33 lesions ≥20mm. Two readers assessed all lesions according to LI-RADS v.2017 criteria and ESGAR consensus statement on liver MR imaging and clinical use of liver-specific contrast agents. Statistical analysis was performed to compare diagnostic ability of both guidelines including receiver operative curves (ROC) and area under curve (AUC).

**Results:**

For LR ≥ 4 sensitivity, specificity, accuracy, and AUC were 96%, 75%, 88.6%, and 85.5, respectively. For LR5 they were 74%, 95%, 80%, and 84.5, respectively. For ESGAR criteria with major and additional features, they were 88%, 75%, 84.3%, and 81.5, respectively. For ESGAR criteria only with major features they were 78%, 80%, 78.6%, and 79, respectively. AUC analysis revealed that overall diagnostic ability of LI-RADS was higher than ESGAR but the results did not show statistical significance.

**Conclusions:**

Both LI-RADS and ESGAR guidelines presented high diagnostic ability in HCC diagnosis of MRI studies with hepatobiliary contrast agents. More complex LI-RADS criteria performed better than ESGAR guidelines and it may justify extra effort that needs to be put in the report. However, the results were not statistically different and the simplicity of the ESGAR guidelines should also be taken into consideration.

## 1. Introduction

Hepatocellular carcinoma (HCC) is the most common primary malignancy of the liver and the second leading cause of cancer death in the world [1]. CT and MR are the imaging techniques that often allow making a definite diagnosis of HCC without a need to biopsy the lesion. HCC is the only tumor that can be diagnosed with imaging only, without a need for histopathological confirmation [2]. A reliable way to diagnose HCC is crucial in the context of high morbidity and mortality [3] and patients diagnosed with HCC at very early stage have more therapeutic options available [4].

There are many guidelines that try to facilitate and unify HCC diagnosis in imaging studies [2], for example, American Association for the Study of Liver Diseases (AASLD) [5], European Association for the Study of the Liver (EASL) [6], and Asian Pacific Association for the Study of the Liver (APASL) [7] guidelines. This study however concentrates on American and European radiological societies guidelines.

The American College of Radiology developed Liver Imaging Reporting and Data System (LI-RADS) [8] to classify lesions in patients with high risk of developing HCC and to stratify this risk into categories. For the purpose of this article we use “LI-RADS” name for LI-RADS CT/MRI classification pertaining to CT and MRI studies. There are also separate classifications for ultrasound surveillance (US LI-RADS) and for contrast enhanced ultrasound studies (CEUS LI-RADS) and they do not play any role in classifying nodules seen on MRI.

There are 5 main categories in LI-RADS: LR1: definitely benign lesions, LR2: probably benign, LR3: intermediate probability of malignancy, LR4: probably HCC, and LR5: definitely HCC. LR5 category is supposed to give almost 100% certainty for a lesion to be HCC without the need of histopathological confirmation. The remaining categories include LR-NC for observations that cannot be categorized due to insufficient image quality, LR-M for malignant lesions but probably not HCC, and LR-TIV for tumor in vein.

LI-RADS classification concerns patients with high risk of developing HCC: patients with cirrhosis OR hepatitis B infection OR current or prior HCC. LI-RADS uses 5 major features: arterial phase hyperenhancement, wash-out appearance, capsule appearance, size, and threshold growth. There are also many ancillary features that can upgrade or downgrade category by one but not above LR4. The most recent update of this classification was published in 2017 [9].

European Society of Gastrointestinal and Abdominal Radiology (ESGAR) published its own guidelines on liver imaging [10]. They include recommendations on how to diagnose HCC on MR examinations.

ESGAR guidelines state that “a focal liver lesion larger than 10 mm in the cirrhotic liver showing wash-in and wash-out (major criteria for HCC diagnosis) should establish a confident diagnosis of HCC and pathologic confirmation is not necessary–statement 44” and “if one of the major criteria is lacking (either wash-in or wash-out), hypointensity on hepatobiliary phase plus restricted DWI or hyperintensity on T2, lesions are highly suspicious for HCC–statement 45” [10].

It is worth noting that both LI-RADS and ESGAR guidelines consider hypointensity of focal hepatic lesion on hepatobiliary phase as additional feature favoring the diagnosis of HCC. Moreover, both guidelines distinguish two specific hepatobiliary contrast agents - in studies performed with gadoxetate acid (Gd-EOB-DTPA, Primovist® or Eovist®, Bayer HealthCare, Berlin, Germany) wash-out can be reported only in portal phase because of an overlap between delayed and hepatobiliary phases [11]. When using gadobenate dimeglumine (Gd-BOPTA, Multihance®, Bracco, Milan, Italy) the wash-out can be reported in both portal and delayed phase.

ESGAR consensus statement on liver MR imaging and clinical use of liver-specific contrast agents [10] has been issued in 2016 but there have been no published data on diagnostic ability of these guidelines. To the best of our knowledge, there have been some studies on LI-RADS efficacy but they have not been compared with ESGAR guidelines.

Therefore, the aim of the study was to compare sensitivity, specificity, accuracy, and overall diagnostic ability of LI-RADS criteria and ESGAR guidelines in HCC diagnosis in MR examinations performed with hepatobiliary contrast agents.

## 2. Methods

The hospital databases (2008-2017) were searched for patients that had HCC or regenerative/dysplastic nodules diagnosed in pathology reports and had MRI with hepatobiliary contrast agent done before the surgery/biopsy. The reverse search was also performed to find all patients with HCCs, regenerative nodules, or dysplastic nodules reported on MRI that had histopathological confirmation. All included patients had high risk of developing HCC. The study included only suspicious lesions that were histopathologically confirmed; none of HCCs or regenerative nodules were reported as LR1, so benign lesions other than regenerative nodules (e.g., cysts or hemangiomas) were not included in the study.

MR studies were done on 1.5T Siemens Magnetom Avanto. The following MR sequences were used: T2 TSE coronal 5mm with fat saturation (fs), T2 TSE axial 5mm fs, T2 TSE dual echo axial, T1 TSE in and out of phase, T2 haste coronal 3mm, DWI (b values: 0, 50, 100, 200, 400, 800, and 1200), ADC, and trueFISP coronal 3mm. The noncontrast enhanced cholangiographic images were acquired using 3D multislice TSE sequence in oblique coronal plane at 0.8mm slice thickness. For 3D multislice TSE sequences multiplanar reconstructions were additionally generated using maximum intensity projection (MIP) algorithm.

Dynamic phase is as follows: 3D T1 GRE (VIBE) before and after contrast (early and late arterial, portal venous, equilibrium, 5-min delayed, and hepatobiliary phase) with TR = 3 ms and TE = 1.1 ms and slice thickness of 3mm.

Two kinds of hepatobiliary contrast were used. Gadobenate dimeglumine (Gd-BOPTA) was used in 6 patients (14 lesions reported) and in 26 patients (56 lesions reported) the studies were performed with gadoxetate acid (Gd-EOB-DTPA). The hepatobiliary phase images were acquired 70 minutes after injection of gadobenate and 20 minutes after injection of gadoxetate.

Restriction of diffusion was reported when the lesion had high signal in DWI (b = 800-1200) and low signal on ADC map.

MRI studies were retrospectively evaluated by 2 radiologists with 5 and 18 years of experience in abdominal imaging. In case of disagreement a consensus was reached. All nodules were evaluated under ESGAR criteria, whether they could be classified as HCCs using major features and all were assigned LI-RADS categories according to LI-RADS v.2017 [9]. Then all ESGAR-negative (lacking at least one of major criteria) lesions >10 mm were assessed for hepatobiliary phase hypointensity, restricted diffusion on DWI and T2-hyperintensity. Lesions >10 mm that were hypointense in hepatobiliary phase (HBP) and showed restriction of diffusion on DWI or hyperintensity on T2-weighted images were reported as highly suspicious for HCC.

Chi2 test and Fisher's exact test were used to compare categorical variables. Univariable and multivariable logistic regression was used to assess odds ratios (OR) and 95% confidence intervals (CI). Receiver operative curves (ROC) and area under curve (AUC) were used to compare diagnosis abilities of the classifiers. DeLong test was used to compare AUC. P value < 0.05 was considered to denote statistical significance. Bonferroni correction was used for multiple comparisons.

There were 4 major groups in the analysis (Table 1):

- LR ≥ 4 meant lesions classified as LR4 or LR5.
- LR5 group included only lesions categorized as LR5.
- “ESGAR major feat.” category in our analysis (Table 1) concerned application of major features (wash-in and wash-out) only. It refers to statement 44 of ESGAR consensus [10] which concerns lesions reported as definitely HCC.
- “ESGAR major +add. feat.” category presents results of application of both major and additional features (HBP hypointensity +T2-hyperintensity and HBP hypointensity+ restricted diffusion) so it includes lesions reported as 100% HCC as well as highly suspicious for HCC. It refers to statements 44 and 45 of ESGAR consensus [10].

## 3. Results

There were 70 lesions included into the analysis from 32 patients (24 males and 8 females). Number of nodules per patient varied between 1 and 8, where 18 (56.3%) patients had only 1 lesion, 5 (15.6%) patients had 2 lesions, 4 (12.5%) patients had 3 lesions, and 5 (15.6%) patients had 4 or more lesions. All nodules were histopathologically confirmed by surgery (n = 67) or core-needle biopsy (n = 3). Fifty lesions were finally diagnosed as HCCs and 20 as regenerative nodules. There were various HCC risk-factors among the patients; however, all of them had cirrhotic livers. Thirteen patients had hepatitis C; 5, hepatitis B; 12, alcoholic liver disease; 1, primary biliary cholangitis; and 1, primary sclerosing cholangitis.

There were 6 lesions <10mm in diameter and 31 lesions of 10-20mm in diameter, and the remaining 33 nodules were larger than 20mm.

Among HCCs there were 2 lesions categorized as LR3, 11 lesions, as LR4 and 37 lesions as LR5. In regenerative nodules group there were 5 LR2 lesions, 10 LR3 lesions, 4 LR4 lesions, and one LR5 lesion. Both LR3 HCCs were 10-19mm and showed arterial phase hyperenhancement without wash-out, capsule, diffusion restriction, or hepatobiliary phase hypointensity. No lesions were associated with tumor in vein.

**Figure 1:**
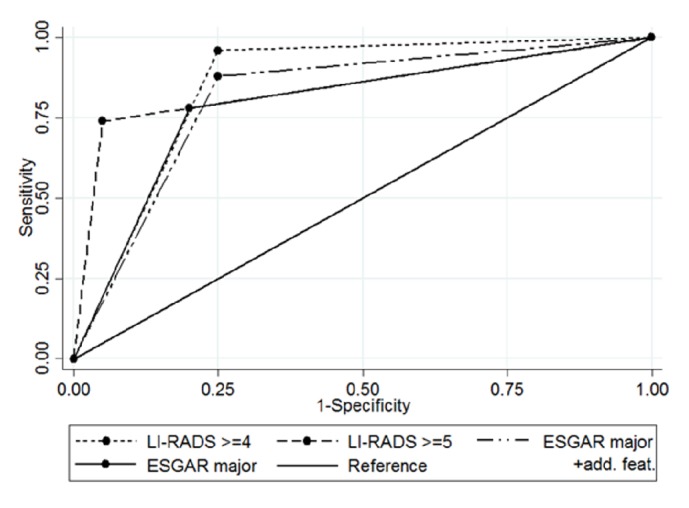
ROC curves for LI-RADS ≥ 4, LI-RADS 5, and ESGAR with major features only and ESGAR after application of additional features.

For lesions LR ≥ 4 the sensitivity was 96%, specificity 75%, and accuracy 88.6%. For lesions LR = 5 sensitivity was 74%, specificity 95%, and accuracy 80%. Positive predictive value (PPV) for LR ≥ 4 was 90.6% and for LR = 5 it was 97.4%. Negative predictive value (NPV) for LR ≥ 4 was 88.2% and for LR = 5 it was 59.4%. AUC for LR ≥ 4 was 85.5 and for LR = 5 it was 84.5 (Table 1 and Figure 1.).

After inclusion of LR3 lesions into analysis the sensitivity for LR ≥ 3 reached 100%; however, specificity dropped to 25%, PPV decreased to 76.9%, and NPV increased to 100%.

There were 43 lesions that were classified as HCCs according to major ESGAR criteria (size > 10mm, wash-in and wash-out). Thirty-nine of them were confirmed pathologically as HCCs, while 4 of them were regenerative nodules. The sensitivity was 78%, specificity 80%, and accuracy 78.6%. After application of additional features (HBP hypointensity +T2-hypointensity or HBP hypointensity + restriction of diffusion on DWI) 49 lesions were reported as HCCs. Among 6 new nodules suspected of being HCC, there were 5 lesions pathologically confirmed as HCCs and one regenerative nodule. There were 6 HCCs (12%) that did not meet ESGAR criteria for HCC diagnosis. After this upgrade sensitivity was calculated to be 88%, specificity was 75%, and accuracy was 84.3%. PPV for major ESGAR criteria was 90.7% and NPV was 59.3%, while after upgrade they were 89.8% and 71.4%, respectively. AUC for ESGAR “major” was 79.0 and for ESGAR “major + add. feat.” it was 81.5 (Table 1 and Figure 1).

The analysis and comparison of AUC in corresponding categories (LR ≥ 4 versus ESGAR major + additional features and LR5 versus ESGAR with major features) showed higher diagnostic ability of LI-RADS criteria (AUC: 85.5 versus 81.5 and 84.5 versus 79.0, respectively) (Tables 2 and 3); however, the differences were not statistically significant.

## 4. Discussion

HCC is the only malignant tumor that can be diagnosed based on imaging features only. There are many guidelines for reporting imaging studies in terms of HCC diagnosis. The perfect criteria would aim for excellent specificity and very good sensitivity. The two most recent of the guidelines, LI-RADS v. 2017 and ESGAR consensus statement on liver MR imaging and clinical use of liver-specific contrast agents, have been compared in this study.

Both LI-RADS and ESGAR criteria showed high diagnostic abilities in HCC diagnosis on MRI with hepatobiliary contrast agents with LI-RADS giving better results in sensitivity, specificity, accuracy, and overall diagnostic ability in corresponding categories.

LI-RADS 3 is an ambiguous category of lesions having similar probability of being malignant and benign. There is no category or statement in ESGAR guidelines that would correspond with LI-RADS 3 in terms of HCC probability. For that reason, LI-RADS 3 was analyzed statistically but without comparison with ESGAR guidelines. Only 4% of HCCs (2 lesions) were categorized as LR3 which was similar to the study by Choi et al. [12] and Tanabe et al. [13] who reported, respectively, 6% and 4% of HCC positive LR3 lesions. A little higher values were reported by Liu et al. [14] (17%) and much higher by Darnell et al. [15] (69%). It is difficult to determine reasons of such differences but different nature of the studies (prospective, ultrasound surveillance based study by Darnell et al. and retrospective in others) should be taken into consideration. Also, Darnell et al. studied lesions smaller than 2cm and they tend to have different imaging features than larger lesions; Liu et al. study included mostly lesions >2cm. In our study the diameters of the analyzed group were more balanced; 31 lesions (44%) were <2cm.

The sensitivity and specificity for LR ≥ 4 were 96% and 75%, while for LR5 it was 74% and 95%, respectively. These are similar results to those in Basha et al. study [16], which showed lower sensitivity for LR5 (67%) though. Very low number (N = 2) of HCCs reported as category LR3 supports LI-RADS as a sensitive test. High (95%) specificity for LR5 lesions is very close to the “100% HCC” target the LI-RADS classification is aiming for.

Our results show that addition of LR4 to LR5 category increased sensitivity (from 74% to 96%) but, as expected, it was followed by decrease in specificity (from 95% to 75%). This specificity impairment supports the need of having separate LR4 and LR5 categories.

There were four LR4 lesions in the regenerative nodules group and 11 in the HCC group. It demonstrates much higher chance for an LR4 lesion to be an HCC than to be a regenerative nodule and it is in concordance with LI-RADS guidelines treating such lesions as having high probability of malignancy.

Addition of LR3 lesions to the analysis increased sensitivity to 100% but it significantly decreased specificity (to 25%). It supports intermediate probability of malignancy of LR3 lesions and such lesions should be followed up or biopsied. The LR3 lesions that were diagnosed as HCCs did not present with any specific imaging features that would be helpful in distinguishing them from benign nodules. They were 10-19mm in size and presented with arterial phase hyperenhancement but no “wash-out”, capsule appearance, and threshold growth. They also showed no ancillary features, e.g., diffusion restriction or hepatobiliary phase hypointensity.

In study by Liu et al. [14] LR5 lesions exhibited higher sensitivity (84.8%) and NPV (80.9%) than in our study and similarly high specificity (95.8%) and PPV (96.8%). For LI-RADS categories 4 and 5, the specificity was higher (88.2%) than in our study with similar sensitivity (93.8%), PPV (92.3%) and NPV (90.5%). The study by Darnell et al. [15] showed much lower sensitivity for LR5 (42.3%) with similar specificity (98.2%). Addition of LR4 category caused an increase in sensitivity (65.3%) with a slight decrease in specificity (96.4%). A study by Yao et al. [17] showed the differences in HCC somatic mutations between populations of Asian and European ancestry, so some differences in appearance on MRI between these populations should also be taken into consideration (all patients in our study were of European ancestry). Another reason could be the size of lesions included in the studies: in our study 44% of lesions were <2cm and Darnell et al. included exclusively lesions <2cm. In Liu et al. study the nodules <2cm in diameter constituted only 20% of LR4 and LR5 lesions (only in these 2 categories the diameters were reported).

In ESGAR consensus on liver imaging there are 2 statements directly addressing MR features of liver nodules in terms of HCC probability. Statement 44 contains major criteria for HCC diagnosis (wash-in and wash-out). For lesions > 10 mm in diameter it should establish a confident diagnosis of HCC and pathologic confirmation is not necessary [10]. This statement corresponds to LI-RADS 5 category in terms of very high confidence of HCC diagnosis. According to statement 45 a lesion lacking wash-in or wash-out but presenting additional features (hypointensity in hepatobiliary phase plus restricted DWI or hyperintensity on T2) can be reported as highly suspicious for HCC. This statement corresponds with LR4 category (probably HCC). The statements 44 and 45 are a part of a complex consensus statement on liver MR imaging and clinical use of liver-specific contrast agents but they have not been directly addressed in the main text of the ESGAR Consensus, so no more detailed information about reporting was mentioned.

In our study the LR ≥ 4 category corresponds to ESGAR with both major and additional features applied and includes lesions reported as HCC or highly suspicious for HCC as in statements 44 and 45. Out of 6 lesions upgraded by inclusion of additional features in ESGAR only one was a regenerative nodule and 5 were pathologically confirmed as HCC. Inclusion of additional features (HBP hypointensity + diffusion restriction or T2 hyperintensity) in ESGAR criteria increased sensitivity from 78% to 88% and decreased specificity from 80% to 75%. It resulted in higher accuracy (84.3%) and larger AUC (81.5).

In corresponding categories LI-RADS performed better than ESGAR. For LR ≥ 4 lesions the sensitivity was higher (96%) than for corresponding ESGAR “major + add. feat.” category (88%) with the same specificity of 75%. This resulted in higher accuracy 88.6% versus 84.3% and AUC 85.5 versus 81.5.The results also show much higher specificity of LR5 category (95%) compared to ESGAR “major” (80%) but lower sensitivity (74% versus 78%). Overall accuracy for LR5 was 80% and for ESGAR “major” 78.6%. It was confirmed in AUC analysis which showed better results for LR5 (84.5) than for ESGAR “major” (79.0). The statistical analysis comparing AUC of LI-RADS and ESGAR criteria in corresponding categories revealed better performance of LI-RADS against ESGAR; however, it was not confirmed by statistical significance. Lack of statistical significance could be caused by a relatively small number of patients in the study and larger multicentered studies may be needed to confirm the results.

Due to increased access to advanced imaging techniques HCCs are often detected at early stage. Accurate diagnosis of small HCCs is very important but often difficult. A study by Forner et al. [18] presents low sensitivity (61.7% for MRI) for lesions < 20mm. In Choi et al. [19] study the cutoff point was set at 1.5cm; the lesions below this threshold less frequently showed MRI features typical for HCC. All classifications systems or guidelines take the lesion's diameter into consideration but with various cutoff points. ESGAR [10] and AASLD [5] recommend diameter of 10 mm as a cutoff point for lesions to be diagnosed as HCC with high level of certainty. In LI-RADS various cutoff points are used to assign categories (<1cm, 1-2cm, and >2cm) depending on presence of arterial hyperenhancement and lesions < 10mm cannot classified as LR5.

In our study there were 6 lesions <10mm (2 HCCs and 4 regenerative nodules) and all of them were correctly diagnosed by LI-RADS and 4 of them by ESGAR. These results also confirm usefulness of LI-RADS classification.

AASLD and EASL have not included hepatobiliary contrast phase in the recommendations due to their perception of a lack of evidence, while both LI-RADS and ESGAR guidelines emphasize the use of hepatobiliary contrast agents and their role in improving sensitivity of MRI examinations [9, 10] as it was suggested by several studies [20, 21] and seems especially significant in small lesions [21]. To the best of our knowledge, this is the first study comparing two guidelines recommending the use of hepatobiliary contrast agents in diagnosis of HCC.

There is a significant difference in complexity between LI-RADS and ESGAR guidelines with the former being more complicated and time-consuming. LI-RADS classification requires usage of tables as well as frequent verification of the findings with the algorithm and guidelines. ESGAR criteria on the other hand are much easier to use since they utilize major features (>10mm size, wash-in and wash-out) with only 3 ancillary features: HBP hypointensity, T2 hyperintensity, and diffusion restriction). The major ESGAR criteria stay in concordance with AASLD and EASL guidelines that also recommend using typical hallmark of HCC (wash-in and wash-out). However, higher diagnostic ability of LI-RADS versus ESGAR may justify extra effort that needs to be put into the report.

Histopathological confirmation is still considered the gold standard in HCC diagnosis [22]. Inclusion of histopathologically confirmed lesions is a limitation as it could cause bias, especially with negative biopsy [22]. However, all three biopsies in the study yielded positive results (HCC) so there was no bias due to sampling error or mistargeting. Also, application of major features resulted in 80% specificity, which is too low to be used as an absolute reference for HCC diagnosis.

There are some other limitations of the study. One of them is a small number of patients which limited strength of the study and statistical significance of some of the results. The number of benign lesions is smaller than HCCs due to retrospective nature of the study which is a limitation as well. Also, the study pertains to MRI studies with hepatobiliary contrast agents and does not include patients examined with CT or MR with extracellular contrast agents. A future direction of research would probably be a prospective, multimodality study with larger number of patients.

## 5. Conclusion

The diagnostic ability of LI-RADS v. 2017 criteria was higher than ESGAR guidelines in HCC diagnosis using MRI with hepatobiliary contrast agents which may justify extra effort that needs to be put into LI-RADS report. However, the difference was not statistically significant. Due to their simplicity and good accuracy ESGAR criteria may also be considered a valuable tool for the differentiation of focal lesions in cirrhotic liver.

## Figures and Tables

**Table 1:** Statistical analysis of LI-RADS scale for different cut-off points and ESGAR.

Cut-off	**Sensitivity**	**Specificity**	**Accuracy**	**AUC**	**PPV**	**NPV**
	% (N) [95%CI]	% (N) [95%CI]	% (N)	PE [95%CI]	% (N) [95%CI]	% (N) [95%CI]

**LR **≥** 4**	96.0 (48/50) [86.3-99.5]	75.0 (15/20) [50.9-91.3]	88.6 (63/70)	85.5 [75.4-95.6]	90.6 (48/53) [79.3-96.9]	88.2 (15/17) [63.6-98.5]

**LR = 5 **	74.0 (37/50) [59.7-85.4]	95.0 (19/20) [75.1-99.9]	80.0 (56/70)	84.5 [76.6-92.4]	97.4 (37/38) [86.2-99.9]	59.4 (19/32) [40.6-76.3]

**ESGAR major + add. feat.**	88.0 (44/50) [75.7-95.5]	75.0 (15/20) [50.9-91.3]	84.3 (59/70)	81.5 [70.8-92.2]	89.8 (44/49) [77.8-96.6]	71.4 (15/21) [47.8-88.7]

**ESGAR major feat.**	78.0 (39/50) [64.0-88.4]	80.0 (16/20) [56.3-94.3]	78.6 (55/70)	79.0 [68.3-89.7]	90.7 (39/43) [77.9-97.4]	59.3 (16/27) [38.8-77.6]

CI = confidence interval; PE = point estimate.

**Table 2:** Comparison of AUC of LI-RADS ≥ 4 and ESGAR major + additional features.

**Variable **	**AUC**		**P-value**
	**PE**	**95**%**CI**	
**LR **≥** 4**	85.5	75.4-95.6	0.331

**ESGAR+**	81.5	71.5-93.4	

CI = Confidence Interval; PE = Point Estimate.

**Table 3:** Comparison of AUC of LI-RADS 5 and ESGAR with major features.

**Variable **	**AUC**		**P-value**
	**PE**	**95**%**CI**	
**LR **≥** 5**	84.5	76.6-92.4	0.228

**ESGAR**	79.0	68.3-89.7	

CI = Confidence Interval; PE = Point Estimate.

## Data Availability

The data used to support the findings of this study are available from the corresponding author upon request.
